# Prevalence and phenotypic findings of pathogenic or likely pathogenic copy number variants in 10,537 pregnancies

**DOI:** 10.1371/journal.pone.0334445

**Published:** 2025-10-28

**Authors:** Shiwei Ren, Wenjing Gu, Ting Liu, Jinyan Yan, Haixin Dong, Chuchu Niu, Lizhu Guo, Huan Guo

**Affiliations:** 1 School of Clinical Medicine, Jining Medical University, Jining, Shandong, China; 2 Department of Clinical Laboratory, Affiliated Hospital of Jining Medical University, Jining, Shandong, China; 3 Department of Obstetrics, Affiliated Hospital of Jining Medical University, Jining, Shandong, China; Shaheed Rajaei Cardiovascular Medical and Research Center: Rajaie Cardiovascular Medical and Research Center, IRAN, ISLAMIC REPUBLIC OF

## Abstract

**Background:**

Pathogenic and likely pathogenic copy number variations (p/lpCNVs) detected through chromosomal microarray analysis (CMA) are crucial for understanding the etiology of birth defects. However, due to incomplete penetrance and variable phenotypic expression, the intrauterine phenotypic characteristics and genotype-phenotype correlations of these variations remain unclear. Therefore, this study aims to explore the prevalence and clinical implications of p/lpCNVs in a large cohort of pregnant women.

**Methods and findings:**

This study retrospectively analyzed 10,537 prenatal diagnostic cases from 2013 to 2022 at the Affiliated Hospital of Jining Medical University. All pregnant women underwent amniocentesis and chromosomal microarray analysis (CMA). Cases were divided into two groups: the CMA group (194 cases) and the karyotype analysis group (259 cases), based on whether CNVs could be detected by traditional karyotype analysis. The primary study outcomes included the incidence of pathogenic or likely pathogenic CNVs, the distribution of variations in specific chromosomal regions, and the correlation between these variations and clinical phenotypes (e.g., cardiovascular abnormalities, developmental delays). Statistical analyses were performed using the chi-square test and the Mann-Whitney U test, with p < 0.05 considered statistically significant. Among 7,663 amniocentesis CMA cases, 453 cases of pathogenic or likely pathogenic CNVs were identified, with 194 cases in the CMA group and 259 cases in the karyotype analysis group. Specific chromosomal regions, such as 22q11.21 and 16p13.11, were associated with clinical phenotypes such as cardiovascular abnormalities and developmental delays. The incidence of pathogenic CNVs was higher in pregnant women with polyhydramnios and those conceived via assisted reproductive technology (ART). The main limitation of this study is the lack of long-term follow-up data on the clinical outcomes of pathogenic CNVs.

**Conclusions:**

This study demonstrates for the first time that chromosomal microarray analysis (CMA) is superior to traditional karyotype analysis in high-risk pregnancies, especially in those with a single clinical indication, by more effectively detecting small copy number variations. Pathogenic CNVs are more likely to cause structural abnormalities, highlighting the stronger association between pathogenic variations and significant phenotypic consequences. Our data also suggest that factors such as assisted reproductive technology (ART) and polyhydramnios may be associated with the occurrence of p/lpCNVs. Future research should focus on clarifying the genotype-phenotype correlations of p/lpCNVs and exploring the potential impact of ART on genetic variations. Long-term longitudinal studies will help deepen the understanding of these variations’ long-term effects on maternal and fetal health, ultimately improving prenatal diagnostics and genetic counseling.

## Introduction

Birth defects, referred to as congenital anomalies, are structural or functional irregularities detected at birth. These anomalies can impair the body’s structure, function, or metabolism, and in severe scenarios, may result in immediate postnatal death or early infant mortality. Worldwide, approximately 8 million newborns are affected by various birth defects annually, which may involve multiple bodily systems including, but not limited to, the heart, brain, lungs, and limbs. The etiologies of birth defects are multifaceted, encompassing genetic factors, environmental exposures, maternal health conditions, and the administration of specific medications during pregnancy. Chromosomal abnormalities are mainly classified as numerical or structural, including deletions, duplications, and other pathogenic genomic variations [1]. Chromosome abnormalities are mainly numerical and structural abnormalities, including deletions, duplications, and other pathogenic genome variations [2]. Such as 1q21.1 recurrent micro-deletion/microduplicaion syndrome, Williams Beuren syn-drome, 16p11.2 microdeletion/microduplicaion syndrome,16p13.11 microdeletion/microduplicaion syndrome, fetuses with central nervous system anomalies, fetal congenital heart defects [3–5]. Therefore, prenatal diagnosis for pregnant women with clinical indications is currently the main technological means for preventing birth defects.

Chromosomal Microarray Analysis (CMA) represents a sophisticated, high-throughput, and high-resolution genetic testing method that significantly enhances prenatal diagnostics. CMA employs thousands of small DNA probes to accurately identify subtle variations within the genome, such as gains or losses of DNA sequences. This high precision is particularly essential for detecting submicroscopic chromosomal imbalances that result in birth defects, typically undetectable by traditional methods. Consequently, CMA offers a more precise diagnostic alternative to conventional karyotyping, which has a resolution constraint of 5–10 Mb [4]. CMA is particularly adept at identifying copy number variations (CNVs), encompassing deletions and duplications of DNA segments. These CNVs can interfere with gene function, leading to developmental abnormalities and diseases, including various severe birth defects. The advancement of CMA has enabled the detection of micro-deletions and duplications as small as 50–100 Kb [6], marking significant progress in identifying pathogenic CNVs linked to a broad spectrum of genetic diseases. This proficiency makes CMA the preferred approach for assessing pregnancies with abnormal ultrasound results or other indicators of potential genetic anomalies, facilitating early diagnosis and management of pregnancies that might be affected by birth defects [1].

Recognizing this, the American College of Obstetrics and Gynecology (ACOG) and other international bodies have recommended CMA as the preferable method in instances of structural abnormalities identified via prenatal ultrasound. This is based on statistics showing that a substantial portion of fetuses with structural abnormalities have clinically significant CNVs detectable by CMA, which are not identifiable by karyotyping alone [7].

Although the pathogenicity and phenotypic characteristics of CNVs have been well understood in postnatal studies [8], the intrauterine phenotypic characteristics of pathogenic or potentially pathogenic copy number variants (CNVs) have been relatively rarely reported due to incomplete penetrance and variable expression [9–11], leading to an unclear correlation between genotype and phenotype associated with these genetic variations. Incomplete penetrance means that some individuals carrying pathogenic genetic variations may not exhibit any clinical symptoms, while variable expressivity refers to the fact that the same genetic variation can range from asymptomatic to severe even within the same family. This variability poses significant challenges for clinical diagnosis and genetic counseling [12]. We systematically collected a series of clinical data from prenatal examination patients, including fetal structural abnormalities detected by ultrasound examination and CNV information detected by CMA. By describing the overall frequency, clinical manifestations, and pregnancy outcomes of pathogenic/likely pathogenic CNVs, we delve into the relationship between CNVs and fetal phenotypic characteristics during the prenatal period, providing references for the prenatal management of pathogenic CNVs.

## Materials and methods

### Ethics statement

This study was approved by the Ethics Committee of the Affiliated Hospital of Jining Medical University, with the ethics review number 2025-04-C002, and the approval date was April 16, 2025. Given the retrospective nature of data collection and the anonymization of patient information, informed consent was not required. This waiver was approved by the Ethics Committee of the Affiliated Hospital of Jining Medical University. The data were accessed for research purposes on April 17, 2025.

### Participant selection and inclusion criteria

From 2013 to 2022, Jining Medical University Affiliated Hospital enrolled 10,537 pregnant women requiring prenatal diagnosis in the study. This retrospective study collected fetal data from the prenatal diagnostic center of the Affiliated Hospital of Jining Medical University. All pregnant women underwent amniocentesis to extract fetal amniotic fluid for CMA testing. Written informed consent was obtained from all participants for the relevant tests. The voluntary acceptance of invasive prenatal diagnosis and CMA testing as the inclusion criteria by the pregnant women was for reasons including: (1) Ultrasound structural abnormalities, encompassing single or multi-system structural anomalies; (2) Soft ultrasound markers: increased nuchal translucency, absent or short nasal bone, ventricular echogenicity, increased bowel echogenicity, ventriculomegaly, widened cisterna magna, choroid plexus cysts, pyelectasis, and short femur; (3) Non-structural abnormalities such as intrauterine growth restriction, polyhydramnios/oligohydramnios; or (4) No ultrasound abnormalities, including women with factors related to assisted reproductive technology (ART), abnormal serological screening results, abnormal non-invasive prenatal testing (NIPT) results, advanced maternal age, a family history of chromosomal abnormalities/previous abnormal pregnancies (FH), history of adverse pregnancy outcomes, and other prenatal diagnostic factors. In this study, pregnant women without apparent clinical indications for invasive prenatal testing were included in the screening process due to factors such as advanced maternal age (≥35 years) and the requests of the patients or their families.

These cases of pathogenic or likely pathogenic CNVs were categorized based on whether the size of the CNVs could be detected by traditional karyotype analysis, resulting in the formation of two groups: the karyotype analysis group and the CMA group. The inclusion criteria for the karyotype analysis group involve CNVs larger than 10 Mb.

A 3-month follow-up was conducted for all cases to monitor the clinical outcomes.

### Sample preparation and CMA

Amniocentesis samples were collected under ultrasound guidance. A 10 ml tube of amniotic fluid was collected and stored at 4°C. The sample was used for CMA testing, and for amniotic fluid samples with maternal cell contamination, the CMA test was conducted after amniotic cell culture. Parents’ peripheral blood of 3 ml was collected when necessary. CMA samples used genomic DNA extracted from amniotic fluid and peripheral blood using the QIAamp DNA Blood Mini Kit (QIAGEN, Germany). CMA was conducted using the Affymetrix CytoScan 750K Array (Affymetrix, USA) chip according to the manufacturer’s instructions. Analysis was performed using ChAS 4.2. All procedures were strictly conducted following the manufacturer’s instructions. CNV analysis was based on the human reference genome 37 (NCBI37hg19) and was evaluated and compared using several databases, including DGV (http://dgv.tcag.ca/dgv), OMIM (https://omim.org/), DECIPHER (https://decipher.sanger.ac.uk/), and PubMed (https://www.ncbi.nlm.nih.gov/pubmed/). These databases provide critical reference information for the clinical significance of CNVs, helping to confirm the pathogenicity or potential clinical impact of the variations.

Quality control measures included probe validation and repeat testing for selected samples to ensure reliability of the CMA results.

The CMA group were classified into three categories: (1) clinically significant, i.e., pathogenic/likely pathogenic CNVs, which are typically closely associated with clinical phenotypes; (2) variants of unknown significance (VOUS), referring to CNVs whose clinical significance has not been determined or those with clinical penetrance below 10%, such as duplications at the 15q13.3, 16p11.2, and 16p11.13 loci [13,14]; (3) normal findings, i.e., no CNVs, benign/likely benign CNVs, or VOUS findings with clinical penetrance below the reported cutoffs of 1Mb for deletions and 2Mb for duplications.

### Statistical analysis

Data analysis was executed using SPSS version 22.0 (IBM Corp., Armonk, NY, USA). The study employed chi-square tests for categorical data to examine the distribution differences of chromosomal anomalies and clinical indications across groups. For continuous variables not following a normal distribution, the Mann–Whitney U test was employed to evaluate disparities in demographic and clinical characteristics. The significance threshold was set at p < 0.05. This analytical method enabled a thorough assessment of the correlation between chromosomal findings identified by CMA and their clinical implications, ensuring robust and statistically valid conclusions.

## Resultes

**Fig 1:**
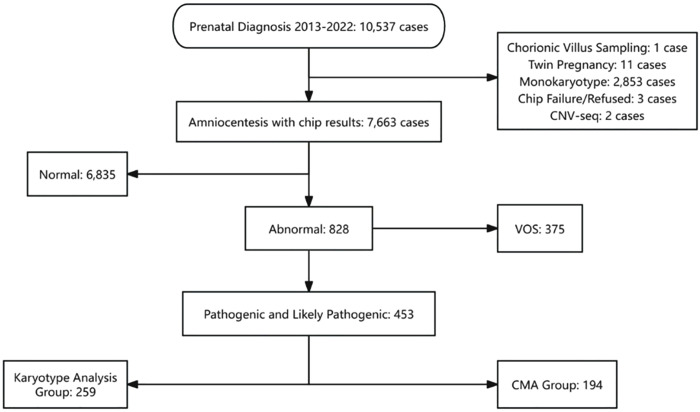
The study process of prenatal diagnosis cases.

A total of 10,537 prenatal diagnosis cases were enrolled from 2013 to 2022. Among the 7,663 cases that underwent CMA testing, 453 (5.9%) were identified with pathogenic or likely pathogenic CNVs. These were subdivided into the karyotype analysis group (n = 259) and the CMA-only group (n = 194). The study workflow and detailed classifications are summarized in Fig 1.

This figure illustrates the workflow of 10,537 prenatal diagnosis cases from 2013 to 2022, showing the categorization into CMA testing, abnormal results, and further subdivision into karyotype-detectable and CMA-only groups.

VOS: Variants of Unknown Significance

CMA: Chromosomal Microarray Analysis

CNV – Copy Number Variation

Of the 453 p/lpCNV cases, 369 were pathogenic and 84 were likely pathogenic. The majority presented with ultrasound abnormalities, particularly soft markers, while a substantial subset (38.6%) showed no ultrasound abnormalities. Table 1 summarizes the detailed distribution of clinical categories and CNV classifications.

**Table 1:** Distribution of pathogenic and likely pathogenic CNVs across different ultrasound abnormality groups and the no ultrasound abnormality group.

Category	Total	PCNVs	LPCNVs
Ultrasound Structural Abnormalities	2 (0.4%)	2 (0.5%)	0 (0)
Soft Ultrasound Markers	88 (19.4%)	77 (20.9%)	11 (13.1%)
Non-structural Abnormalities	4 (0.9%)	3 (0.8%)	1 (1.2%)
No Ultrasound Abnormalities	175 (38.6%)	140 (37.9%)	35 (41.7%)
Two or More of the Above Conditions	184 (40.6%)	147 (39.9%)	37 (44.1%)
Total	453	369	84

### Analysis of demographic characteristics

The demographic characteristics of the karyotype and CMA groups are summarized in Table 2. Maternal age was comparable between groups, but the proportion of women aged ≥35 years was significantly higher in the karyotype group (42.5% vs. 27.9%, p = 0.001). No significant difference was observed in mean gestational age.

**Table 2:** Demographic characteristics and abnormal CMA findings in the study.

Characteristics	Karyotype Analysis Group (n = 259)	CMA Group (n = 194)	p value
Maternal age (years)	31.25 ± 8.72	30.24 ± 6.05	0.148
≥ 35 years old	110/259 (42.5%)	54/194 (27.8%)	0.001 (10.287)
Gestational ages (weeks)	19.87 ± 1.96	20.40 ± 2.85	0.453
No clinical indications	0/259 (0)	1/194 (0.5%)	0.247 (1.338)
1 clinical indications	51/259 (19.69)	75/194 (38.66)	0.000 (19.877)
2 clinical indications	88/259 (33.98)	75/194 (38.66)	0.304 (1.056)
3 clinical indications	65/259 (25.10)	34/194 (17.53)	0.054 (3.723)
4 clinical indications	29/259 (11.20)	6/194 (3.09)	0.001 (10.218)
5 clinical indications	19/259 (7.34)	3/194 (1.55)	0.005 (8.046)
6 clinical indications	5/259 (1.93)	0/194 (0)	0.052 (3.787)
7 clinical indications	1/259 (0.39)	0/194 (0)	0.386 (0.751)
8 clinical indications	1/259 (0.39)	0/194 (0)	0.386 (0.751)
structural anomalies	15/259 (5.79)	9/194 (4.64)	0.588 (0.294)

p value: comparisons were made between the Karyotype Analysis Group and CMA Group

The distribution of clinical indications between the two groups is shown in Table 2. Cases with one indication were significantly more frequent in the CMA group, while cases with four or five indications were significantly more frequent in the karyotype group (all p < 0.01). No significant differences were observed for other categories.

**Fig 2:**
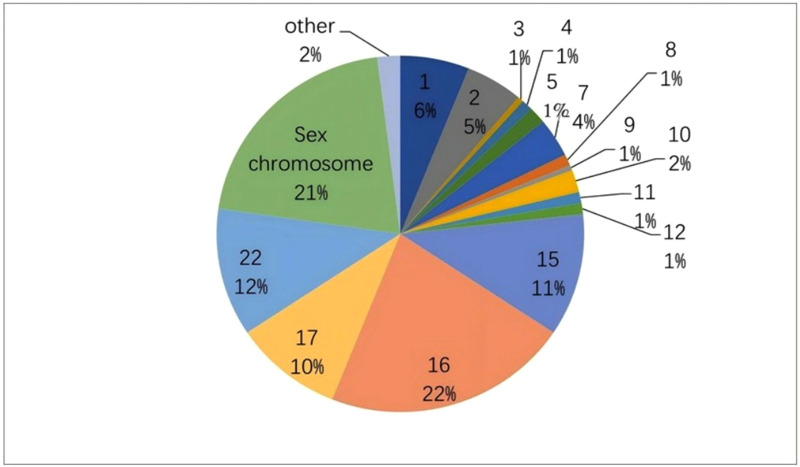
The chromosomal distribution of pathogenic/likely pathogenic copy number variations (p/lpCNVss) in the CMA Group (n = 194).

In the CMA group (n = 194), pathogenic or likely pathogenic CNVs were most frequently located on chromosomes 16, 22, and 1. Variants on chromosomes 15 and 17 were also relatively common, while most other chromosomes accounted for less than 5% each. No p/lpCNVs were identified on chromosomes 6, 14, 19, or 20 (Fig 2, Table 3).

**Table 3:** The chromosomal distribution of microdeletion and microduplication regions.

Chromosome number	number	Microdeletion Regions (Number)	Microduplication Regions (Number)
1	12	1q21.1-q21.2 (5), 1p36.33-p36.32 (1)	1q21.1-q21.2 (5) 1p36.33-p36.32 (1)
2	10	2q37.3(2);2q23.1(1);2q23.2-q24.1(1); 2p16.3(1);2q13(1);2q11.2(1)	2p25.1-p24.3(1);2q11.1-q11.2(1); 2q13(1)
3	1	3p22.1-p21.31(1)	
4	2	4q34.1-q34.3(1); 4p16.3-p16.2(1)	
5	1		5q23.2(1)
7	7	7q11.23(2)	7q11.23(5);
8	2	8p23.3-p23.2(2)	
9	1	9p24.3-p24.2(1)	
10	4	10q21.3(1);10q26.3(1);10q11.22-q11.23(1); 10p15.3(1)	
11	2	11p14.1-p13(1),	11p15.5-p15.4(1)
12	2	12p13.33(1); 12q21.31-q22(1)	
15	21	15q11.2(18);15q13.2-q13.3(1)	15q13.3-q14(1);15q11.2-q13.1(1)
16	42	16p12.2(3);16p11.2(5); 16p13.3(1); 16p13.11(19)	16p11.2(10); 16p13.11(2); 16p13.3(1);16q24.1-q24.3(1)
17	19	17q12(5);17p13.3-p13.2(1); 17p12(4)	17p13.3(1);17q12(3);17p12(4); 17q11.2(1)
22	23	22q11.21(5)	22q11.21(18)
Sex Chromosomes	41	Xp22.33(2);Xp22.31(26);Xp21.1-p21.2(6);Xq26.3-q27.3(1);Xq21.31-q21.33(1); Yq11.21-q11.22(1)	Xq28(4)
Other	4		

The “other” category includes cases where each patient has two or more pathogenic microdeletion or microduplication regions.

This pie chart presents the chromosomal distribution of pathogenic/likely pathogenic CNVs in the CMA group (n = 194). Chromosome 16 accounts for the highest proportion of abnormalities (22%), followed by chromosome 22 (12%) and chromosome 1 (6%). Notably, no pathogenic CNVs were detected on chromosomes 6, 14, 19, and 20. The figure highlights key chromosomal regions affected by CNVs, underscoring their relevance in prenatal diagnosis.

### CMA group

In the CMA group, several recurrent microdeletion and microduplication regions were identified. The most common pathogenic regions included Xp22.31, 22q11.21, 16p13.11, 15q11.2, and 16p11.2. Additional recurrent regions were observed at 1q21.1-q21.2, 17p12, and 17q12, among others (Table 4).

**Table 4:** The number of occurrences of microdeletions and microduplications detected by CMA in specific chromosomal regions.

Microdeletion Regions	Number(132)	Microduplication Regions	Number(64)
Xp22.31	26	22q11.21	18
16p13.11	19	16p11.2	10
15q11.2	18	1q21.1-q21.2	5
Xp21.1–21.2	6	7q11.23	5
1q21.1-q21.2	5	17p12	4
16p11.2	5	Xq28	4
17q12	5	17q12	3
22q11.21	5	16p13.11	2
17 p12	4	15q11.2	1
16p12.2	3	1p36.33-p36.32	1
7q11.23	2	2p25.1-p24.3	1
8p23.3-p23.2	2	2q11.1-q11.2	1
Xp22.33	2	2q13	1
2q37.3	2	5q23.2-1	1
1p36.33-p36.32	1	11p15.5-p15.4	1
2q23.1	1	15q13.3-q14	1
2q23.2-q24.1	1	15q11.2-q13.1-1	1
2p16.3	1	16p13.3-1	1
2q13	1	16q24.1-q24.3-1	1
2q11.2	1	17p13.3-1	1
3p22.1-p21.31	1	17q11.2-1	1
4q34.1-q34.3	1		
4p16.3-p16.2	1		
9p24.3-p24.2	1		
10q21.3	1		
10q26.3	1		
10q11.22-q11.23	1		
10p15.3	1		
11p14.1-p13	1		
12p13.33	1		
12q21.31-q22	1		
15q13.2-q13.3	1		
16p13.3	1		
17p13.3-p13.2	1		
Xq26.3-q27.3	1		
Xq21.31-q21.33	1		
Yq11.21-q11.22	1		

### Clinical manifestations and outcomes family verification results

**Fig 3:**
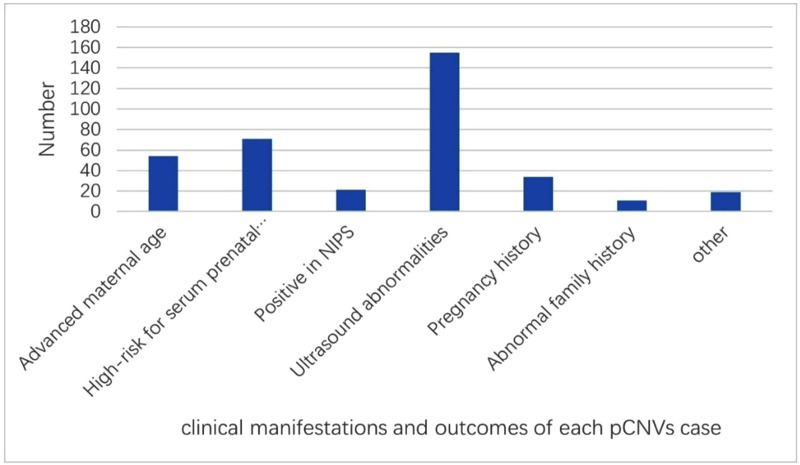
Clinical manifestations and outcomes of each p/lpCNVs case.

The clinical manifestations of p/lpCNV-positive cases were heterogeneous. The most common indications were ultrasound abnormalities (n = 155), abnormal maternal serum screening (n = 69), and advanced maternal age (n = 54). Among ultrasound findings, soft markers were more frequent than structural anomalies. Other contributing factors included abnormal obstetric or family history and assisted reproductive technology (ART). Detailed distributions are presented in Fig 3 and Table 5.

**Table 5:** The distribution of different clinical phenotypes and pregnancy outcomes associated with p/lpCNVs detected by CMA.

	Chromosomes involved with p/lpCNVs																		
chromosome	16	X	22	15	17	1	2	7	10	5	4	8	11	12	3	9	Y	other	total
total	42	40	23	21	19	12	10	7	4	1	2	2	2	2	1	1	1	4	194
Advanced maternal age	12	10	7	7	5	2	4	2	0	0	0	1	2	1	0	0	1	0	54
High-risk for serum prenatal screening	12	19	7	8	5	5	3	1	4	0	1	0	0	1	0	0	0	3	69
Positive in NIPS	2	5	5	1	2	1	1	0	1	0	0	1	0	0	1	1	0	0	21
Ultrasound abnormalities	35	31	25	19	16	9	5	8	2	0	0	1	1	0	0	1	1	1	155
Soft markers	33	31	19	19	16	9	5	8	2	0	0	1	0	0	0	1	1	1	149
Increased NT/NF	6	4	3	5	1	4	0	3	0	0	0	0	0	0	0	0	0	0	26
Persistent left superior vena cava	0	0	1	0	0	0	0	0	0	0	0	0	0	0	0	0	0	0	1
Single umbilical artery	0	0	1	1	1	0	0	0	0	0	0	0	0	0	0	0	0	0	3
Echogenic fetal bowel	2	4	2	1	1	0	0	1	0	0	0	0	0	0	0	0	0	0	11
Echogenic intracardiac focus	8	8	2	3	4	2	1	0	2	0	0	1	0	0	0	0	1	1	33
Renal echo enhancement	0	1	0	0	3	0	0	0	0	0	0	0	0	0	0	0	0	0	4
Choroid plexus cyst	10	5	0	3	5	0	1	0	0	0	0	0	0	0	0	0	0	0	24
Ventriculomegaly	0	1	2	1	0	0	0	1	0	0	0	0	0	0	0	0	0	0	5
Slender Cavum septum pellucidum	0	1	1	0	0	0	1	0	0	0	0	0	0	0	0	0	0	0	3
Hydronephrosis	3	4	4	0	0	0	0	1	0	0	0	0	0	0	0	0	0	0	12
hypoplastic nasal bone	2	0	0	1	1	0	0	0	0	0	0	0	0	0	0	0	0	0	4
tricuspid regurgitation	2	1	1	1	0	1	1	0	0	0	0	0	0	0	0	1	0	0	8
hypoplastic phalanx	0	0	0	0	0	1	0	0	0	0	0	0	0	0	0	0	0	0	1
Low-lying conus medullaris	0	0	0	1	0	0	0	0	0	0	0	0	0	0	0	0	0	0	1
Absent 12th ribs12	0	0	0	1	0	0	0	0	0	0	0	0	0	0	0	0	0	0	1
aortic arch anomalies	0	0	0	1	0	0	0	1	0	0	0	0	0	0	0	0	0	0	2
Unfilled gallbladder	0	1	0	0	0	0	0	0	0	0	0	0	0	0	0	0	0	0	1
pulmonary valve thickening	0	0	0	0	0	0	0	1	0	0	0	0	0	0	0	0	0	0	1
persistent right umbilical vein	0	1	0	0	0	0	0	0	0	0	0	0	0	0	0	0	0	0	1
retrograde venous A wave	0	0	0	0	0	1	1	0	0	0	0	0	0	0	0	0	0	0	2
Pleural effusion	0	0	1	0	0	0	0	0	0	0	0	0	0	0	0	0	0	0	1
Nasolacrimal duct cyst	0	0	1	0	0	0	0	0	0	0	0	0	0	0	0	0	0	0	1
Structural defects	2	0	6	0	0	0	0	0	0	0	0	0	1	0	0	0	0	0	9
Cardiovascular abnormalities	0	0	3	0	0	0	0	0	0	0	0	0	0	0	0	0	0	0	3
Renal abnormalities	0	0	2	0	0	0	0	0	0	0	0	0	0	0	0	0	0	0	2
Intrauterine growth retardation	1	0	2	0	0	0	0	0	0	0	0	0	1	0	0	0	0	0	4
intestinal obstruction	1	0	0	0	0	0	0	0	0	0	0	0	0	0	0	0	0	0	1
Pregnancy history	7	9	1	2	2	0	6	2	0	1	1	1	0	0	0	0	0	0	32
Abnormal family history	4	4	1	0	0	0	1	0	0	0	0	0	0	0	1	0	0	0	11
Assisted reproduction	5	2	0	1	2	1	0	0	0	0	0	0	0	0	0	0	0	0	11
Others (polyamniotic fluid, ovulation promotion)	0	0	0	2	2	1	0	0	1	0	0	0	0	0	0	0	0	1	7
Pregnancy outcomes																			
TOP	27	20	14	16	11	7	6	6	4	1	2	2	2	1	1	0	1	4	125
Be born	13	20	9	5	7	5	4	1	0	0	0	0	0	1	0	1	0	0	66
Loss to follow-up	2	0	0	0	1	0	0	0	0	0	0	0	0	0	0	0	0	0	3

**NIPS**: Non-Invasive Prenatal Screening;

**NT/NF**: Nuchal Translucency/Nuchal Fold

**TOP**: termination of pregnancy

**Ultrasound Structural Abnormalities Detected by CMA**

This figure summarizes the clinical indications and pregnancy outcomes of the 194 p/lpCNVs cases detected by CMA. Ultrasound abnormalities, high-risk serum prenatal screening, and advanced maternal age were the most common clinical indicators. The pregnancy outcomes show that 124 cases resulted in termination, 66 cases resulted in live births, and 3 cases were lost to follow-up.

More than one indication may be observed in each case; thus, the total number of cases in different indications was more than the total cases (n = 194).

In our study, we identified multiple ultrasound structural abnormalities in 194 pregnant women using CMA, as detailed in Table 6. The primary pathogenic regions identified were 22q11.21 and 16p12.2. The main structural abnormalities detected included ventricular septal defect, cardiac malformation, renal structural abnormalities, growth restriction, and intestinal obstruction.

**Table 6:** Distribution of Ultrasound Structural Abnormalities Detected by CMA in 194 Pregnant Women.

Structural abnormalities	Number	CMA group
Ventricular septal defect, cardiac malformation.	2	arr[GRCh37] 22q11.21(18648855_21800471)x1; arr[GRCh37] 22q11.22q11.23(22997928_24995256)x3
Growth restriction.	3	arr[GRCh37] 22q11.21q11.23(21804596_25179302)x3; arr[GRCh37]16p11.2(28763849_29051191)x1,16p12.2(21841353_22442007)x1 arr[GRCh37] 11p15.5p15.4(230680_5183175)x3
Renal abnormalities.	2	arr[GRCh37] 22q11.21(18648855_21800471)x1 arr[GRCh37] 22q11.21(18,919,478_21,461,017)x3
Intestinal obstruction.	1	arr[GRCh37]16p13.11(14,892,976_16,524,976)x1,16p12.2(21,841,354_22,442,007)x1
Ventricular septal defect, growth restriction.	1	arr[GRCh37] 22q11.21(18648855_21800471)x1

Specifically, two cases exhibited both ventricular septal defect and cardiac malformation, associated with pathogenic variations in the 22q11.21 region. Three cases presented with growth restriction, linked to abnormalities in the 22q11.21, 16p11.2, and 11p15.5 regions. Renal structural abnormalities were observed in two cases, both involving the 22q11.21 region. Additionally, one case of intestinal obstruction was associated with pathogenic variations in the 16p13.11 and 16p12.2 regions. Another case showed both ventricular septal defect and growth restriction, also related to the 22q11.21 region.

We included cases with balanced translocations in our karyotype analysis. There were two cases where the karyotype showed a balanced translocation, but the results of pathogenic CNVs (pCNVs) were not associated with this balanced translocation.

Among the 194 cases of copy number variations (CNVs), family validation and follow-up data were available for 67 cases. Of these, 127 cases did not undergo family verification. A genetic inheritance study was performed on the 67 cases with family validation. Among them, 40 CNVs were maternally inherited, 10 were paternally inherited, and 17 were de novo variations.

All cases underwent a 3-month follow-up, with 194 cases from the CMA group analyzed. Of these, 125 cases resulted in termination, 66 cases resulted in live births, and 3 cases were lost to follow-up.

## Discussion

In our comprehensive study involving 10,537 pregnant women undergoing prenatal diagnosis, we provide a unique and panoramic overview of the prevalence and clinical indications of p/lpCNVs across all systems. This stands in contrast to most existing reports, which typically focus on specific systems such as the nervous, cardiovascular, or renal systems. By offering a broad view of p/lpCNVs, our research underscores their overall impact and importance in prenatal diagnostics. Utilizing Chromosomal Microarray Analysis (CMA), we highlight the broader clinical relevance of p/lpCNVs, particularly in cases with various ultrasound-detected abnormalities. This holistic approach emphasizes the necessity of considering all potential systems affected by p/lpCNVs in prenatal diagnosis.

In our cohort, 453 of 7,663 CMA-tested cases (5.9%) harbored pathogenic or likely pathogenic CNVs (p/lpCNVs); within these, CMA-only positives accounted for 194 cases (2.5% of 7,663). By comparison, Kang et al. analyzed 7,078 samples and reported 83 (1.8%) pathogenic recurrent CNVs detected by CMA [3]. In our study, CMA detected a higher proportion of pathogenic or likely pathogenic CNVs, potentially due to differences in the inclusion criteria for pregnant women. This discrepancy may also reflect differences in study populations. In addition, our study applied broader inclusion criteria covering a wider spectrum of prenatal indications and had a larger CMA-tested sample size, which may have increased the detection of clinically significant CNVs. Additionally, data from Table 2 showed that older pregnant women (≥35 years) are more likely to have chromosomal abnormalities detected by karyotype analysis. In the karyotype analysis group, 110 out of 259 (42.8%) were detected, compared to 54 out of 194 (27.8%) in the CMA group, with a p-value of 0.001, indicating a significant difference. This may be because older pregnant women have a higher risk of chromosomal abnormalities due to large segmental structural and numerical chromosomal abnormalities. This finding aligns with Milone et al.‘s study, which showed that older pregnant women are more likely to have chromosomal abnormalities, often associated with severe phenotypic characteristics [15]. In our study, chromosomal microarray analysis (CMA) and traditional karyotype analysis revealed differences in detecting chromosomal abnormalities under various clinical indications. The data show that with one clinical indication, the detection rate of the CMA group (75/194, 38.7%) was significantly higher than that of the karyotype analysis group (51/259, 19.7%), with a p-value of 0.000. However, for cases with two or three clinical indications, the detection rates between the CMA group and the karyotype analysis group did not show significant differences.This indicates that CMA has a clear advantage in cases with fewer clinical indications. For these cases,CMA is recommended as it may reveal particularly significant findings. Our study shows that particularly for high-risk pregnant women, CMA provides important supplementary information in S1 and S2 Files for cases with fewer or no clinical indications. In situations where there are no clinical indications or only one clinical indication, CMA technology demonstrates a clear advantage.

Our study provides a detailed overview of the chromosomal abnormalities found in our cohort. Notably, chromosomes 16, X, and 22 exhibit higher frequencies of abnormalities. These results align with known genomic hotspots, which are prone to recombination and structural rearrangements that can result in various developmental disorders. For example, abnormalities in chromosome 22, particularly in the 22q11.21 region, are linked to DiGeorge syndrome, which is characterized by cardiac defects, facial anomalies, and immune deficiencies. The high incidence of these abnormalities underscores the importance of targeted analysis of these chromosomal regions during prenatal screening. Fetuses with 22q11.2 deletions typically show abnormal prenatal ultrasound findings, most commonly cardiovascular abnormalities, and may also exhibit gastrointestinal, genitourinary abnormalities, or isolated ultrasound markers [3,16,17]. Our analysis identified several chromosomal hotspots prone top p/lpCNVs, including regions Xp22.31, 22q11.2, 16p13.11, and 1q21.1, with the Xp22.31 region showing the highest frequency in our data. These findings differ from those of Kang et al., who reported high incidence rates in these regions, especially the 22q11.2 region, which had the most common p/lpCNVs across 7,078 studied cases and was significantly associated with fetal ultrasound anomalies [3]. The repeated identification of certain regions as hotspots highlights their susceptibility to genetic rearrangements that can lead to developmental disorders, underscoring the need for specific attention to these regions during prenatal screening to improve the detection of potential genetic disorders. Understanding the clinical significance of p/lpCNVs in these hotspots is therefore crucial. At the same time, differences between our findings and those of Kang et al. may be partly attributable to cohort composition and study design. Our cohort included a broader range of prenatal diagnostic indications, whereas Kang’s study may have recruited more cases with specific ultrasound anomalies, particularly those associated with 22q11.2 deletions. Kang et al. observed a distinct pattern of clinical manifestations associated with these variants, particularly in the 22q11.2 and 16p13.11 regions, often correlating with cardiovascular and neurological anomalies [3]. This underscores the need to integrate chromosomal microarray analysis (CMA) with detailed ultrasound examinations to improve diagnostic accuracy and enable early intervention strategies. At the same time, our findings, while also identifying these regions as important hotspots, revealed somewhat different distribution frequencies and associated clinical features, highlighting the heterogeneity of genotype–phenotype correlations across cohorts, which may result from differences in clinical inclusion criteria and study design rather than a single factor. Ultrasound abnormalities, including soft markers and structural anomalies, are important indicators of chromosomal abnormalities. According our study, the main soft markers detected include echogenic intracardiac focus, increased nuchal translucency (NT), and choroid plexus cysts. Although echogenic intracardiac focus and choroid plexus cysts are common in the general population and are not necessarily pathogenic. Our study also shows that these soft markers do not appear in isolation, suggesting they may not be directly linked to pathogenic CNVs. However, absent or hypoplastic nasal bone and increased renal echogenicity, which appeared with one clinical indication, may be related to pathogenic CNVs and require attention.

In our study, we found that among the 18 cases with ultrasound structural abnormalities, 17 were classified as pathogenic CNVs (pCNVs) and 1 as likely pathogenic CNV (lpCNV). This suggests that pathogenic CNVs are more likely to cause structural abnormalities, highlighting the stronger association between pathogenic variations and significant phenotypic consequences. Additionally, the proportion of soft ultrasound markers was 20.9% in pathogenic CNVs and 13.1% in likely pathogenic CNVs, indicating that pathogenic CNVs are more likely to cause clinical phenotypic abnormalities. This reinforces the observation that pathogenic CNVs have a greater impact on clinical outcomes compared to likely pathogenic CNVs.

Based on our follow-up case data, although no clinical phenotypes were observed, it is important to recognize that some conditions may not manifest until later, necessitating continued monitoring. Further follow-up is essential to gain a more comprehensive understanding of the long-term clinical outcomes of these patients, as some issues may emerge over time. The findings from family validation and follow-up are crucial for genetic counseling, offering valuable insights into the inheritance patterns and the potential clinical risks associated with these CNVs. Additionally, in a separate, unpublished study, we analyzed the copy number variations in fetuses of parents carrying balanced translocations and found that the parental balanced translocation does not affect the copy number variation in the fetus. The importance of this longitudinal data will be emphasized in future studies to enhance the understanding of the implications of pathogenic and likely pathogenic CNVs and to improve clinical decision-making.

The structural abnormalities observed in this study indicate specific correlations with certain chromosomal regions. The ultrasound manifestations of the 22q11.2 region primarily include cardiovascular abnormalities, renal abnormalities, and growth restriction. This is consistent with the findings of Meiying Cai et al., who reported that copy number abnormalities in the 22q11.2 region mainly present as cardiovascular malformations, renal malformations, and isolated ultrasound markers [18]. Pathogenic CNVs on chromosome 16 are mainly associated with intestinal malformations and growth restriction, which aligns with the findings of Wendy K. Chung et al. in their study on 16p11.2 deletion syndrome [19]. There was also one case with a pathogenic CNV in the 11p15.5-p15.4 region that exhibited growth restriction. Other pathogenic CNVs did not show structural abnormalities on ultrasound. These findings further support the association between specific chromosomal regions and structural abnormalities.

NIPS is recommended for screening common trisomies 21, 18, and 13, with studies showing it has higher sensitivity and specificity, reduced need for invasive procedures, and early detection capabilities. Despite its advantages in accuracy, safety, and lower false-positive rates [20], its ability to completely replace serum screening remains uncertain. In our study, serum screening detected 71 cases of pathogenic CNVs, while NIPS detected 21 cases. This observation emphasizes that both serum screening and NIPS have their respective roles in detecting CNVs, and it is important to consider the strengths and limitations of each method when interpreting results.

Additionally, our study found 11 cases of p/lpCNVs in fetuses conceived via assisted reproductive technology (ART), raising concerns about whether ART may increase the risk of p/lpCNVs. We hypothesize that ART technology may lead to CNVs, which is consistent with previous research, However, further studies with larger sample sizes are needed to validate this hypothesis. We also found that in the “Others” category, there were six cases of polyhydramnios, suggesting it may serve as a clinical indication of pathogenic CNVs. Additionally, there was one case of ovulation induction, resulting in a total of seven cases of p/lpCNVs. Furthermore, data from Table 5 in our study indicates that a history of adverse pregnancy outcomes may be associated with an increased detection rate of pathogenic CNVs. Previous adverse outcomes, such as miscarriages or congenital anomalies in prior pregnancies, seem to predispose current pregnancies to similar risks. Whether these factors are directly related to the occurrence of p/lpCNVs also requires further research and data support. In conclusion, our research not only identifies the association between various soft markers and structural anomalies with p/lpCNVs but also emphasizes the potential influence of ART and other prenatal factors on the occurrence of p/lpCNVs.

Despite our study offering a detailed overview of the prevalence and clinical indications of p/lpCNVs in prenatal diagnostics, there are still several limitations. Firstly, due to incomplete penetrance and variable expressivity, the phenotypes of most pathogenic CNVs can vary significantly among individuals. Our ability to link the detected p/lpCNVs with future developmental and clinical outcomes is inadequate. Additionally, pregnant women with fetuses showing multiple clinical symptoms often opt to terminate the pregnancy, leading to a smaller sample size. The relatively small sample size of specific subgroups, such as fetuses conceived through assisted reproductive technology (ART), may also affect the generalizability of our findings regarding the potential impact of ART on the incidence of p/lpCNVs. Further large-scale, longitudinal studies are needed to address these limitations and provide clearer insights into the clinical significance of p/lpCNVs in prenatal diagnostics.

Our research systematically gathered clinical data from patients undergoing prenatal examinations, resulting in an extensive analysis of the prevalence and clinical indications of prenatal panoramic p/lpCNVs. By detailing the overall frequency, clinical manifestations, and pregnancy outcomes associated with p/lpCNVs, we investigated the connection between these chromosomal variations and fetal prenatal phenotypic characteristics. The findings from our study provide valuable insights for the prenatal management of pathogenic CNVs and establish a foundation for enhancing prenatal screening and diagnostic strategies.

This study has several limitations. First, as a retrospective analysis, inherent biases in data collection and case selection could not be fully avoided. Second, long-term follow-up information was incomplete, which limited our ability to comprehensively evaluate the prognostic impact of p/lpCNVs. Third, being a single-center study, the findings may not be fully generalizable to populations with different demographic or genetic backgrounds. Future multicenter prospective studies with extended follow-up are needed to validate and expand upon our results.

## Conclusion

This research provides a comprehensive examination of pathogenic and likely pathogenic copy number variations (p/lpCNVs) identified via chromosomal microarray analysis (CMA) in a large sample of Chinese pregnancies. Our results indicate considerable diversity in the phenotypes and outcomes linked to prenatal p/lpCNVs, highlighting the complexity of genetic diagnostics in prenatal care. The increased number of p/lpCNVs observed in pregnancies complicated by polyhydramnios and those conceived via assisted reproductive technology (ART) underscores the importance of heightened vigilance and specialized care for these groups.Regarding non-invasive prenatal screening (NIPS), while it is highly recommended for screening common trisomies 21, 18, and 13, and has shown higher sensitivity, specificity, and reduced need for invasive procedures, our study suggests that it cannot completely replace traditional serum screening. In our study, serum screening detected 71 cases of pathogenic CNVs, while NIPS detected only 21 cases, emphasizing that both methods have their respective roles in prenatal diagnostics. It is crucial to consider the strengths and limitations of each screening method to ensure optimal results.

CMA has proven to be more effective than traditional karyotype analysis in detecting p/lpCNVs, especially in cases with minimal clinical indications, thus offering crucial insights for prenatal genetic counseling. Our findings suggest that pathogenic CNVs are more likely to result in structural abnormalities, highlighting the stronger link between pathogenic variations and substantial phenotypic consequences. This underscores the importance of early detection and thorough genetic counseling for patients with pathogenic CNVs. Identifying specific chromosomal regions like Xp22.31, 22q11.21, 16p13.11, and 15q11.2 as hotspots for p/lpCNVs provides valuable information for targeted screening and diagnosis.

Our research supports incorporating CMA into routine prenatal diagnostic protocols, especially for high-risk pregnancies and those with subtle or no clinical indications. Future studies should further clarify the genotype-phenotype correlations of p/lpCNVs and explore the potential impact of ART on genetic variations. Longitudinal studies tracking affected pregnancies to postnatal outcomes would offer deeper insights into the clinical significance of detected p/lpCNVs.

In summary, the application of CMA in prenatal diagnostics has significantly improved the detection and understanding of p/lpCNVs, enhancing genetic counseling and pregnancy management. We hope our research findings will help optimize prenatal screening strategies and ensure the health of both mothers and their children.

## Supporting information

S1 File Balanced translocation case revised: Provides data on balanced translocation cases, including gestational age, karyotype, and CMA results. (XLSX)

S2 File CMA results revised: Contains the results data for all cases detected by chromosomal microarray analysis (CMA). (XLSX)
